# Supplementary material for: Complexome profiling of the Chlamydomonas psb28 mutant reveals TEF5 as an early PSII assembly factor
Source: Plant Cell. 2025 Mar 17;37(6):koaf055. doi: 10.1093/plcell/koaf055 (PMC12164590; doi:10.1093/plcell/koaf055)
Supplement: koaf055_Supplementary_Data [file koaf055_supplementary_data.zip › psb28_interactive.zip!/plots/intensity/qualityPlots/peptides/NDA3_Cre05.g232200.t1.2.html]

### Cre05.g232200.t1.2

Trivial name: NDA3  
SeqCoverage [median]: 23.00  
Mass: 77.30 kDa  
  
 

| Sequence | MissedCleavage | Mass [Da] | wtIntensity | muIntensity | Score |
| --- | --- | --- | --- | --- | --- |
| APATAPAR | 0 | 753.41 | 1077.09 | 2121.02 | 124.37 |
| ATEALESSGVEVR | 0 | 1346.67 | 13842.74 | 14668.21 | 250.70 |
| ATPLDKEAPPGGAK | 1 | 1350.71 | 7789.29 | 5925.98 | 95.63 |
| AVVAVGEQPATFGVK | 0 | 1471.80 | 11676.20 | 24915.13 | 161.93 |
| EGAEATEVR | 0 | 960.45 | 9822.56 | 7807.02 | 138.78 |
| EVSDAVALR | 0 | 958.51 | 1971.58 | 3520.50 | 91.32 |
| FELASLPGTSEEDRK | 1 | 1677.82 | 0.00 | 1980.64 | 33.20 |
| MNVEADKLAMR | 1 | 1276.63 | 3491.93 | 4030.09 | 64.44 |
| QQEQLVPGDNGR | 0 | 1339.65 | 0.00 | 655.27 | 159.83 |
| RPQFEIQYDK | 1 | 1322.66 | 0.00 | 1197.66 | 101.93 |
| SLEEEELKAR | 1 | 1202.61 | 4689.00 | 1947.09 | 94.30 |
| VHSESLTK | 0 | 899.47 | 1005.55 | 4079.42 | 141.52 |
| VIDTDTYDVVVVSPR | 0 | 1676.86 | 3204.18 | 9307.89 | 205.46 |
| VVEITINK | 0 | 914.54 | 6303.21 | 6760.67 | 104.45 |
|  |  |  | 64873.3 | 88916.6 |  |
